# Measurements of CFTR-Mediated Cl^−^ Secretion in Human Rectal Biopsies Constitute a Robust Biomarker for Cystic Fibrosis Diagnosis and Prognosis

**DOI:** 10.1371/journal.pone.0047708

**Published:** 2012-10-17

**Authors:** Marisa Sousa, Maria F. Servidoni, Adriana M. Vinagre, Anabela S. Ramalho, Luciana C. Bonadia, Verónica Felício, Maria A. Ribeiro, Inna Uliyakina, Fernando A. Marson, Arthur Kmit, Silvia R. Cardoso, José D. Ribeiro, Carmen S. Bertuzzo, Lisete Sousa, Karl Kunzelmann, Antônio F. Ribeiro, Margarida D. Amaral

**Affiliations:** 1 BioFIG - Centre for Biodiversity, Functional and Integrative Genomics; Faculty of Sciences, University of Lisboa, Lisboa, Portugal; 2 Department of Genetics - National Institute of Health, Lisboa, Portugal; 3 Gastrocentro - Endoscopy Unit - State University of Campinas, Campinas, Brazil; 4 Pediatrics Department - State University of Campinas, Campinas, Brazil; 5 Faculty of Medical Sciences - State University of Campinas, Campinas, Brazil; 6 CIPED - Research Center in Pediatrics - State University of Campinas, Campinas, Brazil; 7 Endoscopy Unit – University Hospital of Campinas, Campinas, Brazil; 8 CEAUL - Center of Statistics and Applications of the University of Lisboa; Department of Statistics and Operation Research, Faculty of Sciences, University of Lisboa, Lisboa, Portugal; 9 Institut für Physiologie – University of Regensburg, Regensburg, Germany; University of Tübingen, Germany

## Abstract

**Background:**

Cystic Fibrosis (CF) is caused by ∼1,900 mutations in the *CF transmembrane conductance regulator* (*CFTR*) gene encoding for a cAMP-regulated chloride (Cl^−^) channel expressed in several epithelia. Clinical features are dominated by respiratory symptoms, but there is variable organ involvement thus causing diagnostic dilemmas, especially for non-classic cases.

**Methodology/Principal Findings:**

To further establish measurement of CFTR function as a sensitive and robust biomarker for diagnosis and prognosis of CF, we herein assessed cholinergic and cAMP-CFTR-mediated Cl^−^ secretion in 524 freshly excised rectal biopsies from 118 individuals, including patients with confirmed CF clinical diagnosis (n = 51), individuals with clinical CF suspicion (n = 49) and age-matched non-CF controls (n = 18). Conclusive measurements were obtained for 96% of cases. Patients with “Classic CF”, presenting earlier onset of symptoms, pancreatic insufficiency, severe lung disease and low Shwachman-Kulczycki scores were found to lack CFTR-mediated Cl^−^ secretion (<5%). Individuals with milder CF disease presented residual CFTR-mediated Cl^−^ secretion (10–57%) and non-CF controls show CFTR-mediated Cl^−^ secretion ≥30–35% and data evidenced good correlations with various clinical parameters. Finally, comparison of these values with those in “CF suspicion” individuals allowed to confirm CF in 16/49 individuals (33%) and exclude it in 28/49 (57%). Statistical discriminant analyses showed that colonic measurements of CFTR-mediated Cl^−^ secretion are the best discriminator among Classic/Non-Classic CF and non-CF groups.

**Conclusions/Significance:**

Determination of CFTR-mediated Cl^−^ secretion in rectal biopsies is demonstrated here to be a sensitive, reproducible and robust predictive biomarker for the diagnosis and prognosis of CF. The method also has very high potential for (pre-)clinical trials of CFTR-modulator therapies.

## Introduction

Cystic Fibrosis (CF), the most common severe autosomal recessive disease in Caucasians, is caused by mutations in the *CF transmembrane conductance regulator* (*CFTR*) gene [1]–[3] which encodes a cAMP-regulated chloride (Cl^−^) channel expressed at the apical membrane of epithelial cells to control salt and water transport [4]. Clinically, CF is characterized by multiple manifestations in different organs, but is dominated by the respiratory disease, the main cause of morbidity and mortality. Airway obstruction by thick mucus and chronic infections, especially by *Pseudomonas aeruginosa* (*Pa*), eventually lead to impairment of respiratory function [5]. Other CF symptoms include pancreatic insufficiency, intestinal obstruction, elevated sweat electrolytes and male infertility [6]. About 1,900 CFTR mutations were reported, but one single mutation (F508del), associated with severe CF, accounts for ∼70% of CF chromosomes worldwide [6]. Despite impressive advances in our understanding of the molecular basis of CF, life expectancy and quality of life for CF patients are still limited [5].

For the vast majority of patients, the diagnosis of classic forms of CF is established early in life and suggested by one or more characteristic clinical features, a history of CF in a sibling or, more recently, by a positive newborn screening result [7], [8]. Such diagnosis is usually supported by evidence of CFTR dysfunction through identification of two CF-disease causing mutations, two abnormal sweat-Cl^−^tests (≥60 mEq/L), and/or distinctive transepithelial nasal potential difference (NPD) measurements [7], [8].

However, depending on the ethnic background of the populations tested [9] there is a fraction of patients escaping such diagnosis criteria by presenting “non-classic” symptoms, i.e., milder disease and often inconclusive evidence of CFTR dysfunction from the available diagnostic tools [10], [11]. For such individuals with clinical phenotypes not fully meeting the CF diagnosis criteria it is also difficult to exclude CF. These are usually described as “CFTR-opathies” [12] or CFTR-related disorders (CFTR-RD) [13], [14]. Moreover, the recently implemented extensive newborn screening programs identify increasing numbers of asymptomatic CF patients merely identified by elevated serum concentrations of immunoreactive trypsinogen (IRT), posing new challenges to the CF diagnosis paradigm [8], [15], [16], especially when associated with borderline sweat [Cl^−^] and/or inconclusive *CFTR* genotypes [10], [11], [15], [16]. To confirm/exclude a CF diagnosis in such increasing numbers of individuals, besides close clinical follow-up, further laboratory support is required [16], in particular, there is a need for robust methods relying on the functional assessment of CFTR.

Assessment of CFTR (dys)function in native colonic epithelia *ex vivo*, as we previously reported, constitutes a good approach to this end [17], [18]. However, since those data were reported, other groups have investigated the abnormalities in electrogenic Cl^−^ secretion in the intestinal epithelium of CF patients using Ussing chamber measurements by different protocols [19]–[21]. Unfortunately, the final composite parameter used by some results from a combination of experimental readouts not all relying on direct measurement of CFTR-mediated Cl^−^ secretion, thus leading to conflicting results and precluding good correlations with clinical symptoms [19]–[21].

Since our previous results have established that quantification of rectal CFTR-mediated Cl^−^ secretion is a sensitive test for the diagnosis and prognosis of CF disease [18], hereunder we applied it to the largest cohort of CF patients and highest number of rectal biopsies ever assessed in a single study analyzing CFTR-mediated Cl^−^ secretion in native tissue *ex vivo* to evaluate its robustness as a diagnosis/prognosis biomarker. Our current data demonstrate significant correlations with CF clinical symptoms, evidencing the value of this method as a superior laboratory tool to support clinical practice. Furthermore, here we also applied this technique to individuals with clinical suspicion of CF to confirm/exclude a CF diagnosis. Through comparison of the values for CFTR-mediated Cl^−^ secretion in these individuals to those both of the CF reference group and of a non-CF control group, we could confirm CF in 16/49 individuals (33%) and exclude it in 28/49 (57%), and five remained with inconclusive diagnosis. Finally, using the Ussing chamber data in statistical discriminant analyses, together with the clinical outcomes and other laboratory measurements, we report that colonic measurements of CFTR-mediated Cl^−^ secretion, alone or in combination with sweat-Cl^−^ and fecal elastase E1 (FEE), provides the best tool for a discriminative diagnosis among patients with Classic CF, Non-Classic CF and non-CF individuals.

## Methods

### Ethics Statement and Subjects

Access to human tissues used in this study received approval from the Research Ethics Committee of the Faculty of Medical Sciences, State University of Campinas (UniCamp, ref. 503/2007). Signed informed consent was obtained from all patients (or parents/tutors, for those <18 yrs). Altogether 524 freshly excised rectal biopsies were analysed from 118 Brazilian individuals, including CF patients with confirmed diagnosis (n = 51), individuals with CF clinical suspicion (n = 49) and age-matched non-CF controls (n = 18) undergoing biopsing with no CF-related disorders and agreeing to participate in the study. Conclusive results were obtained for 113/118 subjects, i.e., 96% of the cases.

### Clinical Assessment

Patient data collected included (Table S1): age at diagnosis, sweat Cl^−^ values (≥2), body mass index (BMI), Shwachman-Kulczycki (SK) scores and presence of meconium ileus (MI). Pancreatic sufficiency (PS) was established by Fecal Elastase E1 (FEE) values (≥2) of ≥200 µg/g stool, while FE concentrations ≥100 and ≤200 µg/g were considered moderate pancreatic insufficiency (PI) and a history of malabsorption together with FEE values <100 µg/g determined PI. Different time point measurements (2–4 per individual) of the best annual forced vital capacity (FVC) and best annual forced expiratory volume in 1 second (FEV_1_) were evaluated from individuals >5–6 years of age, and expressed as a percentage of predicted normal values for sex, age, and height. Additional clinical features included nasal polyposis, glucose intolerance, diabetes, osteopenia, osteoporosis, hepatic involvement and lung pathogens. Criteria for glucose intolerance and diabetes were as defined by the American Diabetes Association [22] by performing the 2h-oral 75g-glucose tolerance test: when glucose levels were 140–199 mg/dL, glucose intolerance was assigned and when above 200 mg/dL diabetes was considered. Criteria for osteopenia and osteoporosis were as defined by the T-score for bone mineral density as recommended by the World Health Organization [23]: osteopenia was assigned for values between −1.0 and −2.5 and below −2.5 it was considered osteoporosis. Regarding the “hepatic involvement” we used previously defined criteria [24] i.e., patients presenting either hepatic steatosis, biliary lithiasis and also chronic liver disease. In most cases, liver disease was initially characterized by hepatic steatosis, and ultimately there was biliary cirrhosis and portal hypertension [24]. Ultrasound exams were used to confirm these clinical findings, analyzing liver parenchyma echogenicity and evidence for portal hypertension [24]. Blood analysis also showed increased values for liver transaminases and γ-glutamil transferase (>1.5 fold at least for 6 months). Presence of lung pathogens was defined by the presence in bronchoalveolar lavage (BAL)/sputum of: *Staphylococcus aureus* (*Sa*), *Pseudomonas aeruginosa* (*Pa*), *Stenotrophomonas maltophilia* (*Sm*), *Burkholderia cepacia* (*Bc*); *Achromobacter xylosoxidans* (*Ax*) or *Aspergillus fumigatus* (*Af*) (see Table S1, under column “Pathogens detected”).

### Measurements of Sweat Cl^−^

Sweat was collected on to pre-weighed Cl^–^-free filter paper for 20–30 min. A minimum sweat rate of 1 g/m2 body surface area/min was required; thus a minimum of 150 mg of sweat was considered adequate. Values for Cl^−^ or Na^+^ concentrations above 160 mmol/l were discarded as not physiological and sweat test repeated. Internal quality control procedures were based on the usage of standard solutions (BioClin™, Pretoria, South Africa) and also samples from individuals with negative and positive sweat tests. The coefficient of variation (CV) was of 15 mmol/l.

### Rectal Biopsies Procedure

Superficial 5–6 rectal mucosa specimens were obtained by biopsy forceps (Endoflex® 3.4 mm, Voerde, Germany) as described before [17], [18], [25], [26] (see Methods S1). One biopsy per individual was histologically analysed to exclude inflammation, haemorrhage, infection or other tissue damage (Fig.S1).

### Ussing Chamber Measurements

Rectal biopsies were analysed in micro-Ussing chambers under open-circuit conditions as established previously (see Methods S1). To minimize sample variability, measurements were performed on 2–5 biopsies (Fig.S2) and data averaged to obtain a single value per individual.

### CFTR Genotyping

Following screening of the 6 most common CFTR-disease causing mutations in the region of Campinas (Brazil) [27]–[29]: F508del, G551D, G542X, R1162X, N1303K, R553X, an extended CFTR mutation search (see Methods S1) was performed when only one/none mutation was found (Table S2, with both traditional and standard nomenclatures [30]).

### Chemicals and Compounds

All chemicals (highest available purity) were from Sigma-Aldrich® (St Louis, MI, USA) or Merck® (Darmstadt, Germany) except for culture media (GIBCO®/Invitrogen, Carlsbad, CA, USA). Fecal Elastase E1 test was from Schebo ® Biotech AG (Giessen, Germany).

### Statistics

For statistical analyses the SPSS software/v.19 (Chicago, IL, USA) was used and a p-value <0.05 was considered statistically significant. Unless otherwise stated, data are mean ± STD (n, number of individuals studied). Details of the statistical analysis for Pearson correlations, Chi-square and discriminant analyses are described under Methods S1.

## Results

### Subjects Under Study and Overview of Clinical Data

The clinical diagnosis of CF was established based on consensus clinical criteria [7], [8], namely: 1) presence of one or more characteristic phenotypic features (chronic sinopulmonary disease; gastrointestinal/nutritional abnormalities; obstructive azoospermia or salt-loss syndrome) and 2) evidence of a CFTR abnormality (increased sweat [Cl^−^] (>60 mEq/L) and/or detection of two CF-disease causing mutations). Using these criteria and the current CF classification terminology [10], two different sub-groups of CF patients (n = 51) were established (detailed clinical data in Table S1): (a) Classic CF patients (n = 46) presenting a severe phenotype and classic disease manifestations (high sweat-Cl^−^; PI; nutrition deficiencies; chronic sinopulmonary disease); and (b) Non-Classic CF patients (n = 5) with an atypical phenotype and showing a milder disease (pancreatic sufficiency-PS; adulthood diagnosis; less serious lung involvement; and at least one organ with CF phenotype).

A third group included individuals with a clinical suspicion of CF (n = 49) of which 27% had only one abnormal sweat-Cl^−^ value; others presented borderline (22%) or normal (20%) sweat-Cl^−^ values (see Methods); and 31% had not been tested for sweat-Cl^−^ at the time. Most of these individuals (69%) had inconclusive genetic testing (20% had only one CF-disease causing mutation identified) and the remainder had not been CFTR-genotyped at the time (Table S1). Most of them (68%) showed mono-symptomatic features, including: respiratory symptoms in 29% (nasal polyps, chronic cough/bronchitis, pneumonia, bronchiectasis or pansinusopathy); 35% had abnormal gastrointestinal signs (nutrients malabsorption; failure to thrive; hepatobiliary disease; chronic diarrhoea; recurrent pancreatitis; diabetes and glucose intolerance; others presented osteopenia/osteoporosis, liver disease or male infertility. Fourteen percent of these “CF suspicion” individuals presented relatively mild lung disease and nutrition abnormalities, one presenting GI and another azoospermia. A group of 9 patients with severe phenotype (both respiratory and gastrointestinal) were initially included in this CF suspicion group (18%) due to their recent identification and while waiting for confirmation of a CF diagnosis (Table S1).

### Assessment of CFTR-mediated Cl^−^ Secretion in Rectal Biopsies

CFTR-mediated Cl^−^ secretion was assessed in rectal biopsies from the above two sub-groups of CF patients (Classic and Non-Classic CF) as CF reference. As non-CF control group, we also analysed CFTR function in age-matched individuals undergoing routine colonoscopy for non-CF related reasons. Finally, measurements were carried out in the CF suspicion individuals to establish/exclude a CF diagnosis, following comparison with values from the CF patients and control groups.

**Figure 1:**
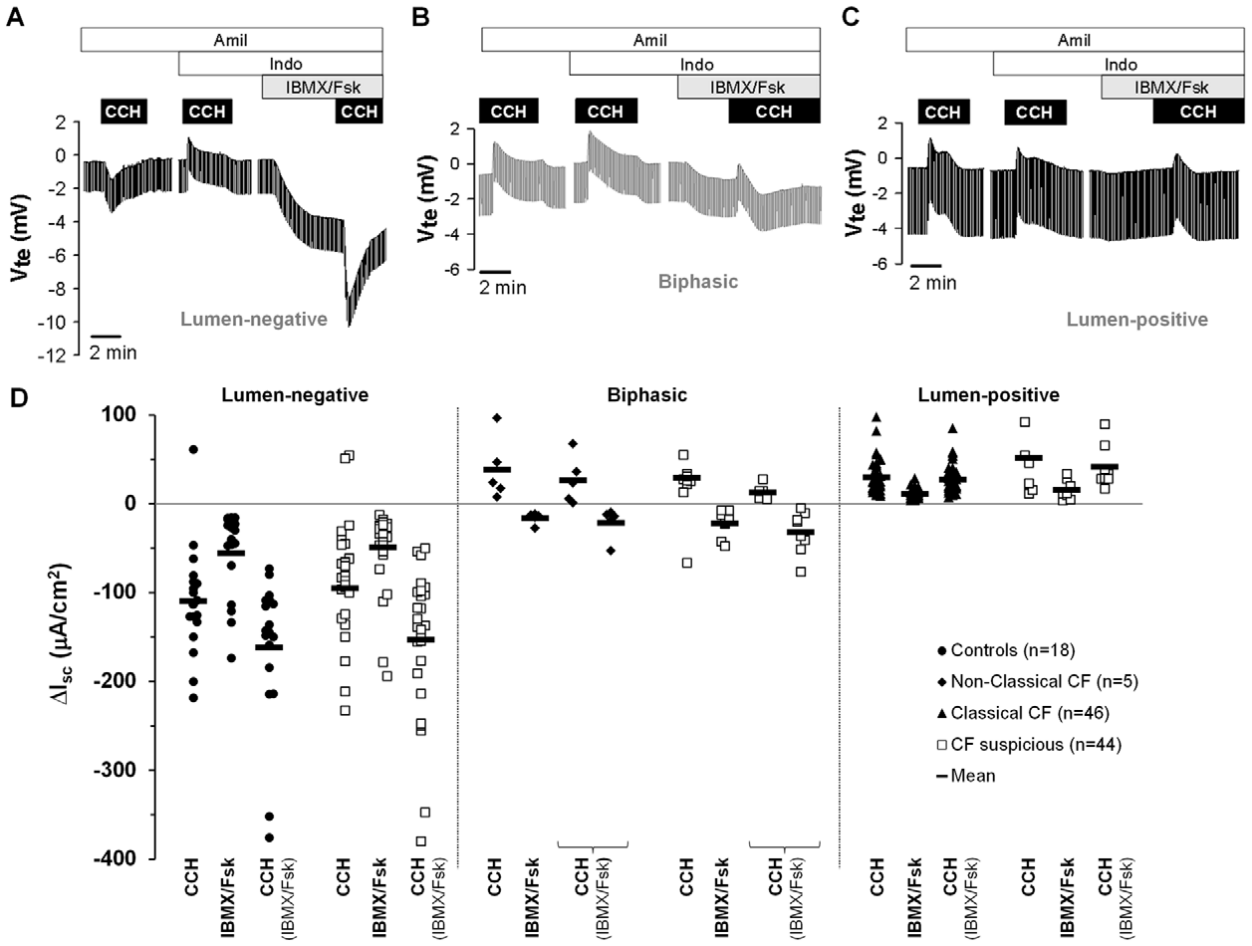
Results from Ussing chamber measurements in rectal biopsies from 113 individuals. Original recordings of the effects of cholinergic (CCH, 100 µM) and cAMP-dependent (IBMX, 100 µM and Fsk, 2 µM, basolateral) activation on transepithelial voltage in rectal tissues from (**A**) a healthy control presenting CCH lumen-negative responses; (**B**) a Non-Classic CF patient showing biphasic responses, thus presenting residual CFTR function; and (**C**) a Classic CF patient with no detectable Cl^−^ secretion, presenting only lumen-positive responses. All the experiments were performed in the presence of Amiloride (Amil, 20 µM, luminal) and Indomethacin (Indo, 10 µM, basolateral). (**D**) Summary of activated short-circuit currents (ΔI_sc_) for basal CCH (I_sc-CCH_), IBMX/Fsk (I_sc-IBMX/Fsk_) and CCH following IBMX/Fsk application (I_sc-CCH(IBMX/Fsk)_) in rectal biopsies from controls (lumen-negative, filled circles, n = 18), Non-Classic CF (biphasic, filled diamonds, n = 5), Classic CF (lumen positive, filled triangles, n = 46) and CF suspicious patients (open squares, n = 44) showing lumen-negative, biphasic and lumen-positive responses. Data represent the mean of the mean measurements on 2–5 rectal biopsies per individual. Black solid line shows mean ΔI_sc_ values for each group represented in µA/cm^2^: Controls (I_sc-CCH_ =  −109.28±14.47; I_sc-IBMX/Fsk_ =  −55.38±11.12; I_sc-CCH(IBMX/Fsk)_ =  −162.07±19.64); Non-Classic CF (I_sc-CCH_ = 47.20±17.86; I_sc-IBMX/Fsk_ =  −16.27±2.83; I_sc-CCH(IBMX/Fsk)_ =  −21.64±8.01); Classic CF (I_sc-CCH_ = 30.13±2.66; I_sc-IBMX/Fsk_ = 11.30±0.78; I_sc-CCH(IBMX/Fsk)_ = 27.17±2.22); and CF suspicious with similar ΔI_sc_ distribution.

As shown previously [17], [18], [25], [26], application of carbachol (CCH) under basal conditions elicited lumen-positive responses in CF and lumen-negative in non-CF tissues (Fig.1A, B, C). Nevertheless, due to variable levels of endogenous prostaglandins, lumen-positive responses can also be observed in non-CF control tissues [17], [26]. Thus, when CCH was applied for a second time, now under indomethacin to completely inhibit endogenous cAMP (and thus CFTR-mediated Cl^−^ secretion), all tissues presented lumen-positive responses correspondent to potassium (K^+^) exiting the cell (Fig.1A, B, C) [17], [26]. Next, when we used 3-isobutyl-1-methylxantine (IBMX) and forskolin (Fsk) to activate cAMP-dependent CFTR-mediated Cl^−^ secretion [17], [18] (I_sc-IBMX/Fsk_), we observed lumen-negative responses in tissues from individuals in both the “non-CF control” group (Fig.1A) and “Non-Classic CF” sub-group (Fig.1B) but lumen-positive responses for those in the “Classic CF” sub-group (Fig.1C).

Finally, and as previously described [17], [18], [25], [26], [31], following stimulation with CCH in the presence of IBMX/Fsk (I_sc-CCH(IBMX/Fsk)_), three different response patterns were observed and quantified in relation to pre-reagent (IBMX/Fsk) baseline values: (i) monophasic lumen-negative (Cl^–^secretory) in tissues from “non-CF controls”, and we quantified such negative peak (Fig.1A); (ii) monophasic lumen-positive (K^+^-secretory) in tissues from the “Classic CF” subgroup, and we determined both peak and plateau (Fig.1C); and finally (iii) biphasic responses, in the “Non-Classic CF” sub-group, and we determined both positive and negative peaks (Fig.1B).

### Confirmation/Exclusion of a CF Diagnosis and Correlation with Genotypes

The equivalent evoked-short-circuit currents (ΔI_sc_) calculated for individuals in the “CF suspicion” group (Fig.1D, open squares, ΔI_sc_ values in Table S1) were compared with the reference values for the CF (“Classic CF” and “non-Classic CF” sub-groups, Fig.1D black triangles and diamonds, respectively) and control groups (Fig.1D, black circles). Accordingly, each individual with a clinical suspicion of CF could be associated with one of these two groups (n = 44): for those with a lumen-negative response (Fig.1A, D) a CF diagnosis was excluded and those presenting only lumen-positive or biphasic responses (Fig.1B, C, D) were considered as CF. For the remainder five, data were inconclusive due to unviability of biopsies. Subsequently, sweat Cl^−^ test and extended genotyping was performed for all CF-suspicious individuals. Positive sweat Cl^−^ and two CF*-*disease causing mutations were eventually detected in all individuals (except one) with absence or residual CFTR-mediated Cl^−^ secretion (see Table S1).

One “CF suspicion” patient with severe CF symptoms and the F508del/S549R genotype, evidenced very low levels of CFTR function (∼5% normalized to controls). In almost all “Classical CF” patients, values for CFTR-mediated Cl^−^ secretion were zero and, in the very rare cases with residual currents these were always lower than 5%. In contrast, for the 28 individuals in the “CF suspicion” group who showed lumen-negative responses, i.e., normal CFTR function (I_sc-CCH(IBMX/Fsk)_ = −153.38±15.33 µA/cm^2^ vs I_sc-CCH(IBMX/Fsk)_ = −162.07±19.64 µA/cm^2^ in the non-CF control group) we could only detect one CF-causing mutation (F508del) in one individual (being thus a CF-carrier) and 2 other mutations in two individuals who were thus compound heterozygous: W1282X/4428insGA and F508del/D1152H, respectively (Table S1). Based on their clinical characteristics (only obstructive azoospermia and bronchiectasis), values of CFTR colonic function (within the normal range) and CFTR genotypes, these two individuals were thus classified as CFTR-RD and both 4428insGA and D1152H were regarded as CFTR-RD mutations. However, the criteria from colon bioelectric measurements do not support *per se* the definition of a group of CFTR-RD subjects, as these individuals have normal colonic CFTR-mediated Cl^−^ secretion.

Overall, based on CFTR-mediated Cl^−^ secretion in the colon and subsequent confirmatory CF diagnostic testing (sweat Cl^−^, *CFTR* genotypes and clinical outcomes (data on Table S1-b), we could classify the individuals in the “CF suspicion:” group (n = 49) as: Classic CF (n = 9), Non-Classic CF (n = 7), CFTR-RD (n = 2) and Non-CF (n = 26). As to the 5 individuals showing inconclusive Ussing chamber measurements, one individual had one CF-disease causing mutation (G542X) and two individuals had RD-related mutations (V562I and G576A). As we could not confirm/exclude CF in these 5 individuals, they were not included in the hereunder correlations.

Functional classification of rarer mutations also results from these analyses, namely (Table S1): 3120+1G>A as class I (2 siblings with 3120+1G>A/R1066C, absence of CFTR-function and severe phenotypes); 1716+18672A>G as class V (2 other siblings with F508del/1716+18672A>G, residual CFTR function −28–34%- and mild CF); I618T as class IV (in a patient with G542X/I618T, 37% CFTR function and mild disease); and L206W as class IV or CFTR-RD mutation (in a patient with F508del/L206W and the highest CFTR function −57%- and very mild disease).

### Correlation between CFTR-mediated Cl^−^ Secretion and Clinical Outcomes

**Figure 2:**
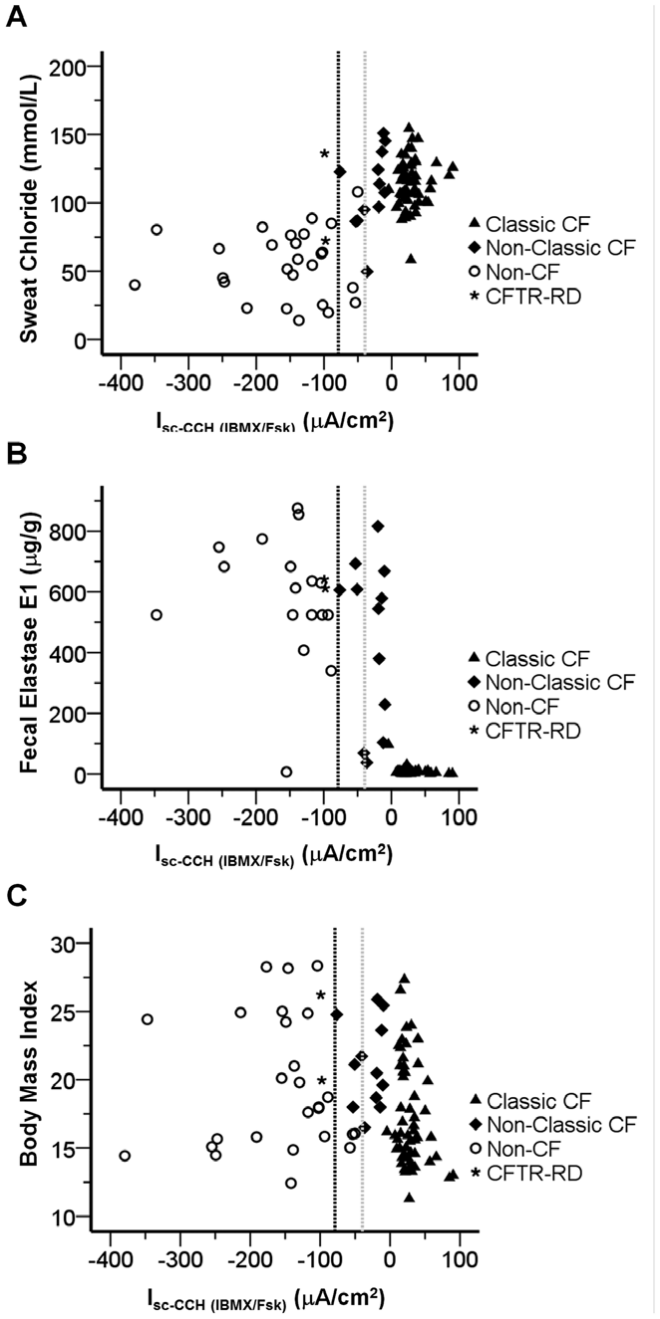
Correlations between CF laboratory measurements and CCH-induced short circuit currents following IBMX/Fsk application (I_sc-CCH(IBMX/Fsk)_). Scatter-plot summarizing the distribution of I_sc-CCH(IBMX/Fsk)_ against (**A**) sweat [Cl^−^] (mmol/l); (**B**) FEE concentrations (µg/g of stools); and (**C**) BMI for all individuals included in the study showing conclusive results (n = 113) and classified according to CF Clinical Diagnosis Consensus Guidelines as: Classic CF (filled triangles, n = 55); Non-Classic CF (filled diamonds, n = 12); CFTR-RD (star, n = 2); and non-CF (open circles, n = 26). Vertical dashed black line represents subtraction of one SD of the mean value calculated for I_sc-CCH (IBMX/Fsk)_ (ΔI_sc_ = −78.77 µA/cm^2^) in non-CF controls (“grey zone”). Vertical dotted grey line represents addition of one SD of the mean value calculated for I_sc-CCH(IBMX/Fsk)_ (ΔI_sc_ = −39.55 µA/cm^2^) in reference sub-group of Non-Classic CF patients.

**Figure 3:**
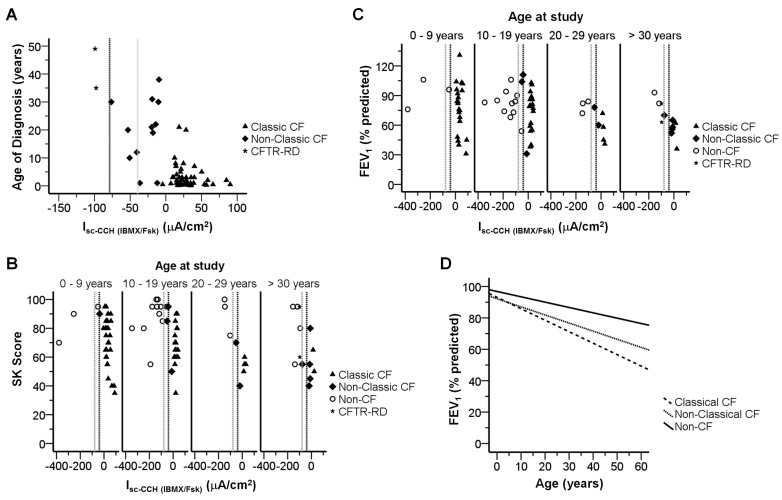
Correlations between CF clinical features and CCH-induced short circuit currents following IBMX/Fsk application (I_sc-CCH(IBMX/Fsk)_). Scatter-plot summarizing the distribution of I_sc-CCH(IBMX/Fsk)_ against (**A**) age at diagnosis (in years); (**B**) SK clinical scores distributed by groups of ages; and (**C**) FEV_1_ distributed by groups of ages. Other details as in Fig.2. (**D**) Mixed regression model for decline rates in *FEV_1_ vs. Age* (n = 232 measurements) for Classic CF (y = 93.15–0.73x), Non-Classic CF (y = 92.23–0.52x), and Non-CF (y = 96.86–0.34×) groups, as described [32], [33].

In order to assess the value of CFTR-mediated Cl^−^ secretion in rectal biopsies as a predictive tool for CF, we attempted to statistically correlate these values with the accepted CF-characteristic parameters (Table 1, see also Methods S1), namely: sweat [Cl^−^] (Fig.2A, S3A); FEE (Fig.2B, S3B); BMI (Fig.2C, S3C, S4A); and age at diagnosis (Fig.3A, S3D). For the correlations involving SK scores (Fig.3B, S3E, S4B) and FEV_1_ (Fig.3C, S3F, S4C), we subdivided patients into 4 age-groups (in years): 0–9; 10–19; 20–29; and ≥30, since these parameters have been shown to decline with age [32].

**Table 1:** Correlations between clinical outcomes and CFTR-mediated I_sc_ in rectal biopsies.

	Assessment of CFTR Function in Rectal Biopsies			
	I_sc-IBMX/Fsk_		I_sc-CCH (IBMX/Fsk)_	
	Pearson(*r*)	p-value	Pearson(*r*)	p-value
**Sweat Chloride**	**+0.495**	**3.41×10^−7^**	**+0.677**	**4.73×10^−14^**
**Fecal Elastase E1**	**−0.721**	**6.83×10^−14^**	**−0.770**	**1.10×10^−16^**
**Age at Diagnosis**	**−0.728**	**1.34×10^−12^**	**−0.713**	**6.18×10^−12^**
**BMI**	−0.169	0.101	**−0.226**	**0.028**
**BMI -** *Age at study*	−0.104	0.319	−0.121	0.244
**SK Score**	**−0.274**	**0.009**	**−0.362**	**4.59×10^−4^**
**SK Score -** *Age at* *study*	**−0.302**	**0.004**	**−0.411**	**6.44×10^−5^**
**FEV_1_**	−0.205	0.071	**−0.240**	**0.035**
**FEV_1_ -** *Age at study*	**−0.251**	**0.028**	**−0.301**	**0.008**

NOTE: Pearson coefficients (r) and p-values showing statistical correlations (*p*<0.05) are highlighted (n = 95).

Our data (Fig.2, 3, S3, S4, Tables 1, S3) show high correlations between CFTR-mediated Cl^−^ secretion values and these parameters describing CF phenotypes. Indeed, patients with absence/very low CFTR-mediated Cl^−^ secretion (≤5%) had higher sweat-Cl^−^ values (114.23±2.33 mmol/l), lower FEE concentrations (9.08±2.04 µg/g), earlier age at diagnosis (2.7±0.6 yrs), lower SK scores (mean_1_ = 71±3, mean_2_ = 69±3; mean_3_ = 56±2; mean_4_ = 58±8), and lower FEV_1_ (mean_1_ = 77±6; mean_2_ = 72±5; mean_3_ = 55±5; mean_4_ = 49±13) than individuals with normal values of CFTR-mediated Cl^−^ secretion. Moreover, CF patients with residual values of CFTR-mediated Cl^−^ secretion had intermediate values in those parameters, namely: sweat-Cl^−^ values (109.80±8.34 mmol/l); FEE concentrations (444.57±78.05 µg/g), age at diagnosis (19.6±3.4 yrs), SK scores (mean_1_ = 90; mean_2_ = 77±14; mean_3_ = 63±12; mean_4_ = 55±7); and FEV_1_ (mean_1_ =  n.a.; mean_2_ = 82±26; mean_3_ = 61±9; mean_4_ = 60±3). Moreover, patients with classic CF and ≤5% CFTR-mediated Cl^−^ secretion consistently presented faster decline rates of pulmonary function [32], [33] (Fig.3-D, dashed line), than Non-Classic CF patients retaining residual (Fig.3-D, dotted line) or normal CFTR functions (Fig.3-D, solid line, *p* = 0.001 by Kruskal-Wallis test). Regarding BMI (Fig.2-C, Table 1), only a modest trend was observed for lower BMI values in CF patients with absence of Cl^−^ secretion, not related to age differences (Fig.S4-A, S3-C, Table 1).

Next, we analysed the distribution of other clinical features (MI, nasal polyposis, diabetes/GI, osteopenia/osteoporosis) and also presence of lung pathogens in the three groups under study. Significant differences (Table 2) were found between the groups under study for the distribution of lung pathogens (*p* = 1.75×10^−6^, 88% of CF patients with pathogens), with predominance of Pa, and MI (*p* = 0.0015, 24% of CF patients with MI).

**Table 2:** Distribution of clinical features among the 3 groups of individuals analysed here: Classic CF; Non-Classic CF and Non-CF.

		CF Clinical Diagnosis			
		Classic CF	Non Classic CF	Non-CF	Total (n)
		I_sc-CCH(IBMX/Fsk)_ = 28.68±2.22 µA/cm^2^; n = 55	I_sc-CCH(IBMX/Fsk)_ = −30.06±6.15 µA/cm^2^; n = 12	I_sc-CCH(IBMX/Fsk)_ = −153.38±15.33 µA/cm^2^; n = 28	
**Lung Pathogens** **(** ***p = *** **1.75×10^−6)^**	Pa	7	3	1	**11**
	Pa withother pathogens	21	5	0	**26**
	Other pathogens	20	3	2	**25**
	Negative	2	0	7	**9**
	n.a.	5	1	18	**24**
**Meconium Ileus (** ***p = *** **0.0015)**	Positive	16	0	0	**16**
	Negative	39	12	26	**77**
	n.a.	0	0	2	**2**
**Nasal Polyposis** (*p* = 0.591)	Positive	10	4	6	**20**
	Negative	44	8	20	**72**
	n.a.	1	0	2	**3**
**Glucose Intolerance (GI)/Diabetes** (*p* = 0.303)	GI	5	1	0	**6**
	Diabetes	5	2	2	**9**
	GI/Diabetes	5	0	0	**5**
	Negative	39	9	23	**71**
	n.a.	1	0	3	**4**
**Osteopenia/Osteoporosis**(*p* = 0.511)	Osteopenia	2	0	2	**4**
	Osteoporosis	6	3	2	**11**
	Negative	46	9	22	**77**
	n.a.	1	0	2	**3**

NOTE: n.a., Not analysed. p-values showing statistical differences among groups under study (*p*<0.05) are highlighted (n = 95). GI, Glucose Intolerance.

Altogether, these data indicate that our approach to measure the level of CFTR (dys)function in rectal biopsies provides data evidencing good correlation with the CF severity.

### Evaluation of the Best Tool for Discriminating CF Patients from Non-CF Individuals

**Figure 4:**
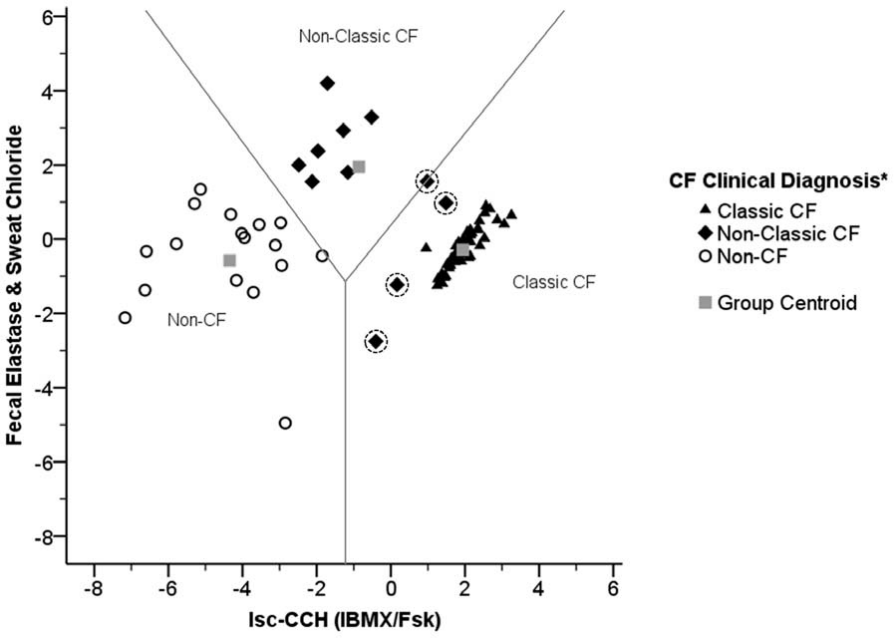
Distribution showing the relative position of each individual in study according to the scores obtained by discriminant analysis (territorial map). Analysis performed for individuals having complete information about I_sc-CCH(IBMX/Fsk)_, FEE concentration in stools and sweat-Cl^−^ (n = 75): Classic CF (filled triangles, n = 47); Non-Classic CF (filled diamonds, n = 11); and non-CF (open circles, n = 17). Misclassified cases are marked with dotted circles. Grey squares represent the group centroids and grey lines the barriers between each group. * according to consensus criteria [7], [8], [10].

Following the above findings, we attempted to establish a CF diagnosis tool which could also serve for disease prognosis. We thus used a stepwise discriminant analysis to evaluate which one(s) among the clinical and laboratory measurements available (sweat-Cl^−^; FEE; BMI; SK score; FEV_1_; I_sc-IBMX/Fsk_; and I_sc-CCH(IBMX/Fsk)_), constitutes the best discriminator factor between patients with Classic and Non-Classic CF and also between these groups and non-CF individuals (see Methods). As shown in Fig.4, discriminant function 1 (x-axis) corresponding to CFTR-mediated Cl^−^ secretion measurements in rectal biopsies under CCH (I_sc-CCH(IBMX/Fsk)_) is the best option to explain the differences between the three groups in 90.4% of cases (Tables S4, S5). Additionally, FEE concentration and sweat-Cl^−^ correspond to discriminant function 2 (y-axis) enabling separation of an additional ∼6% of the remainder individuals (Tables S4, S5). Notwithstanding, four Non-Classic CF patients were still misclassified as Classic CF (Fig.4, black diamonds with dotted circles). Furthermore, to translate this discriminant analyses into a diagnosis algorithm, we have obtained its respective Fisher’s linear classification functions as:

***Classic CF*** = 0.317(Sweat Chloride) +0.005(Fecal Elastase) +0.012(I_sc-CCH(IBMX/Fsk)_) –18.909.

***Non-Classic CF*** = 0.338(Sweat Chloride) +0.028(Fecal Elastase) −0.015(I_sc-CCH(IBMX/Fsk)_) –28.485.

***Non- CF*** = 0.202(Sweat Chloride) +0.026(Fecal Elastase) −0.102(I_sc-CCH(IBMX/Fsk)_) –23.414.

These allow classification of a new CF suspicion case by replacing in these 3 equations the values from laboratory measurements obtained for a given individual. The function giving the highest value will correspond to the CF classification group best describing the individual.

## Discussion

The wide spectrum of CF phenotypes, high variability of CF lung disease, the uncertain (dys)function of many rare CFTR mutations together with increasing numbers of asymptomatic patients identified in recent newborn CF screens, have posed major challenges to clinicians for the establishment of CF diagnoses and prognosis [8], [10], [11]. Such hurdles make it difficult for caregivers to provide adequate genetic counselling and medical care, risking worsening of symptoms and organ damage.

### Good Correlations between CFTR-mediated Cl^−^ Secretion and CF Parameters

To evaluate the robustness of colonic CFTR-mediated Cl^−^ secretion as a diagnosis/prognosis biomarker and thus help overcoming such difficulties, we assessed CFTR (dys)function *ex vivo* in 524 rectal biopsies from 118 individuals, including the largest cohort of CF patients ever analysed by this approach (n = 51), a non-CF (control) group (n = 18) and individuals with clinical CF suspicion to confirm/exclude a CF diagnosis (n = 49). The functional data, demonstrating good correlations with most CF-defining parameters, have also provided key information to adjust the clinical judgment of a CF diagnosis and prognosis.

Our approach consists in direct measurements of colonic CFTR function assessed through both cAMP-dependent and cholinergic Cl^−^ secretion which, as previously shown [25], [26], [31], are strictly dependent on the presence of functional CFTR. Our data here show that CFTR-mediated Cl^−^ secretion is absent or present at almost undetectable levels (<5%) in 244 biopsies from patients with classic forms of CF (Fig.1, Table S1), as defined by consensus criteria [7], [8], [10] including: early age at diagnosis (2.7±0.6 yrs), very high sweat-Cl^−^ (114.23±2.33 mmol/l) and PI (9.08±2.04 µg/g) (Fig.2, 3, S3, S4, Table S3). This group of Classic CF patients also presented other severe CF symptoms (as defined in Methods), like MI (29%), associated diabetes and/or GI (27%), lung pathogens (87%) and also hepatic involvement (29%) (Tables 2, S1). In contrast, a group of patients with milder symptoms, classified as Non-Classic CF by established guidelines [7], [8], [10], evidenced residual CFTR-mediated Cl^−^ secretion (10 to 57%), consistently with our previous data [18]. Indeed, most of patients in this group were diagnosed at an older age (19.6±3.4 yrs) and were PS (444.57±78.05 µg/g) (Fig.2, 3, S3, S4, Table S3). Additionally, by stratifying patients into four different age groups, our functional data also showed good correlations with lung function (FEV_1_) and SK scores, where patients with highest values evidenced residual colonic CFTR-mediated Cl^−^ secretion (Table 1, *r* = −0.301 and −0.411, respectively).

### Comparison with Other Bioelectric Methods

By applying a different Ussing chamber protocol to bioelectric measurements in rectal biopsies, other authors used an overall parameter resulting from a combination of different readouts, some relying on indirect activation of CFTR and thereof proposed a cut-off value [20]. Yet, application of such protocol in another study reported a classic CF patient evidencing readout values higher than the cut-off [21]. So, it is our conviction that such cut-off value cannot be clearly established for usage among different laboratories [20], [21]. Moreover, several technical aspects differed between the current and such protocol. Firstly, here we use bicarbonate (HCO3^−^)-free buffer solutions to exclude a possible contribution of electrogenic HCO3^−^ secretion to lumen-negative V_te_/I_sc_ [18], [34], [35]. Secondly, our measurements evidence stable baselines in contrast to the drifting baselines reported in those studies where agonist responses have to be determined from estimated baselines [21]. Thirdly, our readout is based on direct CFTR-mediated Cl^−^ secretion under complete inhibition of endogenous cAMP [17], [18], [25], [26], [31], [35], instead of complex bioelectric responses involving two different Ca^2+^-dependent agonists and an anion-transporter inhibitor following incomplete prostaglandin inhibition [19]–[21]. Our protocol has the major advantage of using continuous perfusion which allows for pairwise examination of agonists. Altogether, these characteristics, together with the demonstration of its extreme sensitiveness (accurate calculation of CFTR activity down to <5%, Table S1), and high reproducibility (very stable baseline currents during the >2 h course of the experiments and similar recordings in different biopsies from the same individual taken on the same day Fig.S2) make the current approach a superior contribution for CF diagnosis and prognosis (Fig.4).

Another technique to support a CF diagnosis is NPD measurements. The outcome of this approach, however, relies on a composite score including both ENaC- and CFTR-mediated responses [36]–[41], where two abnormal NPD recordings on separate days evidence CFTR dysfunction [7], [8]. The technique, however, has variable applicability for adults and children [41]. Moreover, this *in vivo* procedure requires patient immobilization and sedation (for children <6 yrs) for at least ½ h [41], while the rectal biopsing only takes ∼10 min allowing for multiple *ex vivo* measurements of different samples from the same individual and lead to conclusive results (96% here) for a highest percentage of individuals than NPD (91% [41]). Moreover, NPD values considerably overlap among CF patients, carriers and non-CF controls [37]–[39], [41]. This may be due to the fact that NPD is not a truly quantitative measurement but relies on the pure measurement of voltages.

### Validation as a Biomarker for CF Diagnosis/Prognosis

Importantly, we show here how assessment of colonic CFTR function was a key tool to exclude CF in 28 individuals (2 of classified as CFTR-RD patients). In fact, despite that the clinical features of these individuals suggested CF, both genotyping and sweat test were inconclusive (Table S1). This leads us to conclude of the importance of this approach for patients in the “grey” zone (Fig.2, 3) for the establishment/exclusion of a final diagnosis of CF or CFTR-RD.

**Figure 5:**
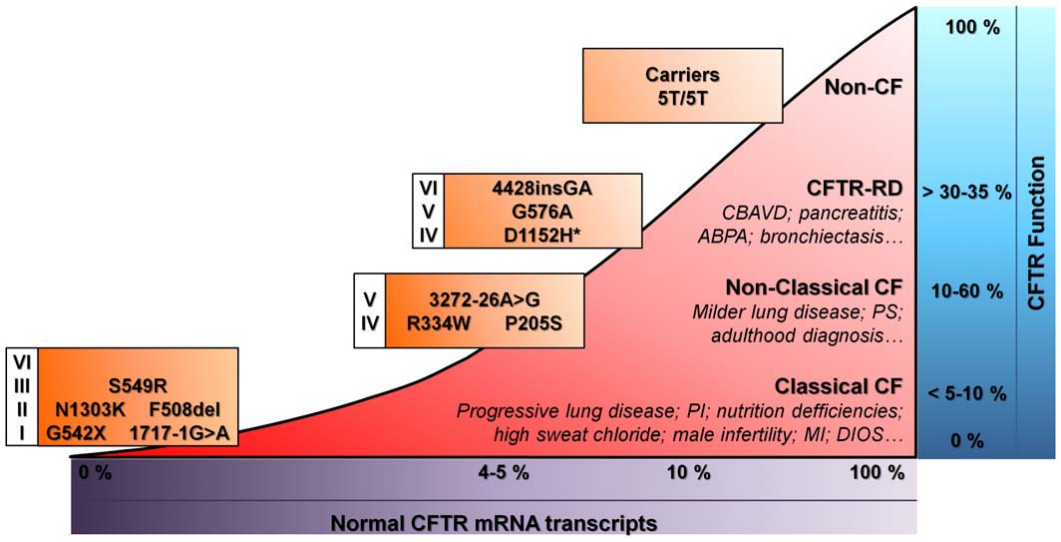
Association of CF clinical phenotypes with the levels of normal CFTR mRNA transcripts, CFTR function (as percentage of maximal CFTR activation normalized to control group) and CFTR mutations (grouped by functional classes of mutations) with examples found in this study. Indicated values are speculative. *D1152H may belong either to CFTR-RD or CFTR-disease causing mutations. CBAVD, Congenital Bilateral Absence of the Vas Deferens; ABPA, Allergic Bronchopulmonary Aspergillosis; PS, Pancreatic Sufficiency; PI, Pancreatic Insufficiency; MI, Meconium Ileus; DIOS, Distal Intestinal Obstruction Syndrome.

Interestingly, our data are also highly informative to correlate CFTR function with *CFTR* genotypes (Fig.5). For instance, we were able to detect very low function (∼5% CFTR function vs non-CF controls) in a patient bearing S549R, recently described as Class III [42], whereas significant CFTR-mediated Cl^−^ secretion was found in two patients bearing 4428insGA and D1152H (84 and 64%, respectively). Thus, we classify the former here as CF-disease causing and the two latter as CF-RD mutations.

In the present study, we have also used a statistical discriminant analysis to identify which clinical and functional parameter(s) better reflects the differences among Classic CF, Non-Classic CF and Non-CF groups for prognosis. Results show that I_sc-CCH(IBMX/Fsk)_ measurements are more discriminative than I_sc-IBMX/Fsk_, evidencing the highest discriminant power (90.4%) among the three groups studied. The second and third parameters with higher discriminant power are FEE and sweat-Cl^−^ concentration, respectively. Using such analysis (Fig.4), one can for example predict that individuals in the “Non-Classic CF” group with values lying closer to the “Non-CF” cluster, have better prognosis than those with values closer to the “Classic CF” cluster. In fact, the only four cases with “Non-Classic CF” which were misclassified as “Classic CF” by this analysis, correspond to individuals with moderate PI and/or low levels of CFTR function. It may, thus be expected that these four patients develop severe CF earlier than most Non-Classic ones. Interestingly, and corroborating such prediction among these four, there is a 19-year old patient with the F508del/G85E genotype, moderate PI (FEE = 103.89 µg/g) and ∼12% of CFTR function (Fig.S2-C, Table S1) who recently (last 1½ yr) started to progress from a relatively mild to moderate-severe lung disease, with five pulmonary exacerbations plus surgery to remove nasal polyps associated with a strong sinusitis.

Recently, we demonstrated that the current approach may be used in pre-clinical assessment of therapeutic compounds efficacy directly on native tissues [43] and similarly it may be used to identify CF patients (and CFTR mutations) who will respond to innovative therapeutic strategies, namely those aimed at increasing the residual CFTR activity and already approved for clinical use for other mutations towards a predictive personalized-medicine approach [18], [43]–[47]. For example, the patient with S549R could be tested *ex vivo* for the correction with the FDA-approved potentiator Ivacaftor, as suggested by the *in vitro* data [42]. Moreover, based on our data showing good correlations of CFTR-mediated Cl^−^ secretion with absolute FEV_1_ values and FEV_1_ decline rate (Fig. 3), as well as taking into account our discriminant analyses (Fig.4) demonstrating that CFTR-mediated Cl^−^ has the highest discriminant power (90.4%) to distinguish among Classic CF, Non-Classic CF and Non-CF groups, we propose that colonic CFTR-mediated Cl^−^ secretion, perhaps together with sweat-Cl^−^, may be the best biomarker in clinical trials aimed at modulating CFTR [44], [48].

### What is the Functional CFTR Threshold to Avoid CF?

Finally, in an attempt to further answer the old question “how much functional CFTR would be enough to avoid CF?” [49] we have put together the current and previous findings, including those concerning mRNA levels, to establish the threshold for CF [18], [50]–[52] (Fig.5). Indeed, we previously showed that ∼5% of normal *CFTR transcripts* (relative to non-CF individuals) is sufficient to attenuate CF severity [50]–[52]. From our current and previous data, we propose that CFTR values above ∼10% of normal *CFTR function* are required for a better CF prognosis since all “Non-Classical CF” patients had values for CFTR-mediated Cl^−^ secretion higher than 10%. Indeed, CF patients with CFTR function below 10% evidence more severe CF (Table S1). Moreover, as non-CF controls and individuals for whom a CF diagnosis was discarded show CFTR-mediated Cl^−^ secretion ≥30–35%, as before [18], we propose to set at 30% the threshold of CFTR function which is necessary to reach to avoid any form of CF disease.

In conclusion, our current approach to measure CFTR-mediated Cl^−^ secretion in rectal biopsies is demonstrated here to be a sensitive, reproducible and robust predictive biomarker for the diagnosis/prognosis of CF. Moreover this method has very high potential to be used as endpoint for (pre-)clinical trials of innovative therapeutic approaches involving CFTR-modulators.

## Supporting Information

Figure S1 **Histological evaluation of rectal biopsies by Hematoxilin-Eosin (HE) and Masson’s Tricome stainings** in control (transversal cut), Non-CF (longitudinal cut), Classical CF (transversal cut) and Non-Classical CF (longitudinal cut) showing a healthy epithelia, namely no fibrotic processes were observed and some biopsies presented inflammatory processes, independent of being CF or not. In HE stained sections we observe nuclei in blue and cytoplasm in pink to red. For Tricome’s Masson we observe collagen in blue, nuclei black, and muscle and cytoplasm in red. Black scale bar represents 250 µM.
(DOCX)

Figure S2 **Original recordings of transepithelial voltage (V_te_) measurements in Ussing chambers obtained in 3–4 rectal biopsies from the same individual evidencing the high reproducibility of the method.** Rectal biopsies from (**A**) Non-CF individual showing large cholinergic (carbachol, CCH, 100 µM, basolateral) and cAMP-dependent (3-isobutyl-1-methylxantine, IBMX, 100 µM, and forskolin, Fsk, 2 µM, basolateral) Chloride (Cl^−^) secretion (lumen-negative responses); (**B**) CF patient homozygous for F508del-CFTR mutation with absence of Cl^−^ secretion (only lumen-positive responses, reflecting potassium (K^+^) secretion, were observed); (**C**) CF patient (genotype: F508del/G85E-CFTR) showing very little (∼12%) cAMP-dependent Cl^−^ secretion (biphasic responses observed upon co-cholinergic stimulation with CCH); and (**D**) CF patient (genotype: 3120+1G>A/L206W-CFTR) presenting larger CFTR residual function (∼57%) and milder phenotype than in (**C**). All the experiments were performed in the presence of Amiloride (Amil, 20 µM, luminal) and Indomethacin (Indo, 10 µM, basolateral). Transepithelial resistance (R_te_) was determined from V_te_ deflections obtained by pulse current injection (0.5 µA).
(DOCX)

Figure S3 **Correlations between CF clinical features and IBMX/Fsk-induced short circuit currents (I_sc-IBMX/Fsk_).** Scatter-plot summarizing the distribution I_sc-IBMX/Fsk_ against (**A**) sweat chloride concentrations (in mmol/l); (**B**) age at diagnosis (in years); (**C**) Body Mass Index distributed by groups of ages; (**D**) Fecal Elastase E1 concentrations (in µg/g of stools); (**E**) Shwachman–Kulczycki clinical scores distributed by groups of ages; and (**F**) FEV1 (% of predicted normal values for sex, age, and height) distributed by groups of ages. Vertical dashed black line represents subtraction of one standard deviation (STD) of the mean value calculated for I_sc-IBMX/Fsk_ in non-CF controls (ΔI_sc_  =  −8.18 µA/cm^2^). Vertical dashed grey line represents addition of one STD of the mean value calculated for I_sc-IBMX/Fsk_ in reference sub-group of Non-Classic CF patients (ΔI_sc_  =  −22.60 µA/cm^2^). Classic CF (filled triangles, n = 55); Non-Classic CF (filled diamonds, n = 12); CFTR-RD (star, n = 2) and Non-CF (open circles, n = 26) individuals.
(DOCX)

Figure S4 **Correlations between CF clinical features and CCH-induced short circuit currents following IBMX/Fsk application (I_sc-CCH (IBMX/Fsk)_).** Scatter-plot summarizing the distribution of I_sc-CCH (IBMX/Fsk)_ against (**A**) Body Mass Index; (**A**) Body Mass Index distributed by groups of ages; (**C**) Shwachman–Kulczycki clinical scores; and (**D**) forced expiratory volume in 1 second (FEV_1_) expressed as a percentage of predicted normal values for sex, age, and height (% predicted).Vertical dashed black line represents subtraction of one standard deviation (STD) of the mean value calculated for CCH induced-ΔI_sc_ following IBMX/CCH application (ΔI_sc_ = −78.77 µA/cm^2^) in non-CF controls. Vertical dashed grey line represents addition of one STD of the mean value calculated for CCH stimulated-ΔI_sc_ following IBMX/CCH application (ΔI_sc_ = −39.55 µA/cm^2^) in reference sub-group of Non-Classic CF patients. Classic CF (filled triangles, n = 55); Non-Classic CF (filled diamonds, n = 12); CFTR-RD (star, n = 2) and Non-CF (open circles, n = 26) individuals.
(DOCX)

Table S1 **Overview of data for all CF patients in the reference group (Table S1a) and in the CF-suspicious group (Table S1b): genotypes, clinical phenotypes and Ussing chamber measurements, leading to confirmation/exclusion of a CF Diagnosis.**
(XLSX)

Table S2 **CFTR mutations found in individuals under study. Gene and protein localization, mutation classification and frequency from the present study are designated. Traditional and HGVS standard nomenclature for CFTR mutations are also indicated.**
(DOCX)

Table S3 **Mean and standard error of the mean values for clinical parameters among the 3 groups of individuals analyzed in this study: Classic CF; Non-Classic CF and Non-CF.**
(DOCX)

Table S4 **Discriminant Functions: Eigenvalues and Wilk’s Lambda statistics.**
(DOCX)

Table S5 **Canonical Discriminant Functions used in the Analysis.**
(DOCX)

Methods S1 **Cl- secretion in Rectal Biopsies.**
(DOCX)
