# Supplementary material for: Measurements of CFTR-Mediated Cl− Secretion in Human Rectal Biopsies Constitute a Robust Biomarker for Cystic Fibrosis Diagnosis and Prognosis
Source: PLoS One. 2012 Oct 17;7(10):e47708. doi: 10.1371/journal.pone.0047708 (PMC3474728; doi:10.1371/journal.pone.0047708)
Supplement: Figure S1 — Histological evaluation of rectal biopsies by Hematoxilin-Eosin (HE) and Masson’s Tricome stainings in control (transversal cut), Non-CF (longitudinal cut), Classical CF (transversal cut) and Non-Classical CF (longitudinal cut) showing a healthy epithelia, namely no fibrotic processes were observed and some biopsies presented inflammatory processes, independent of being CF or not. In HE stained sections we observe nuclei in blue and cytoplasm in pink to red. For Tricome’s Masson we observe collagen in blue, nuclei black, and muscle and cytoplasm in red. Black scale bar represents 250 µM. (DOCX) [file pone.0047708.s001.docx]

**
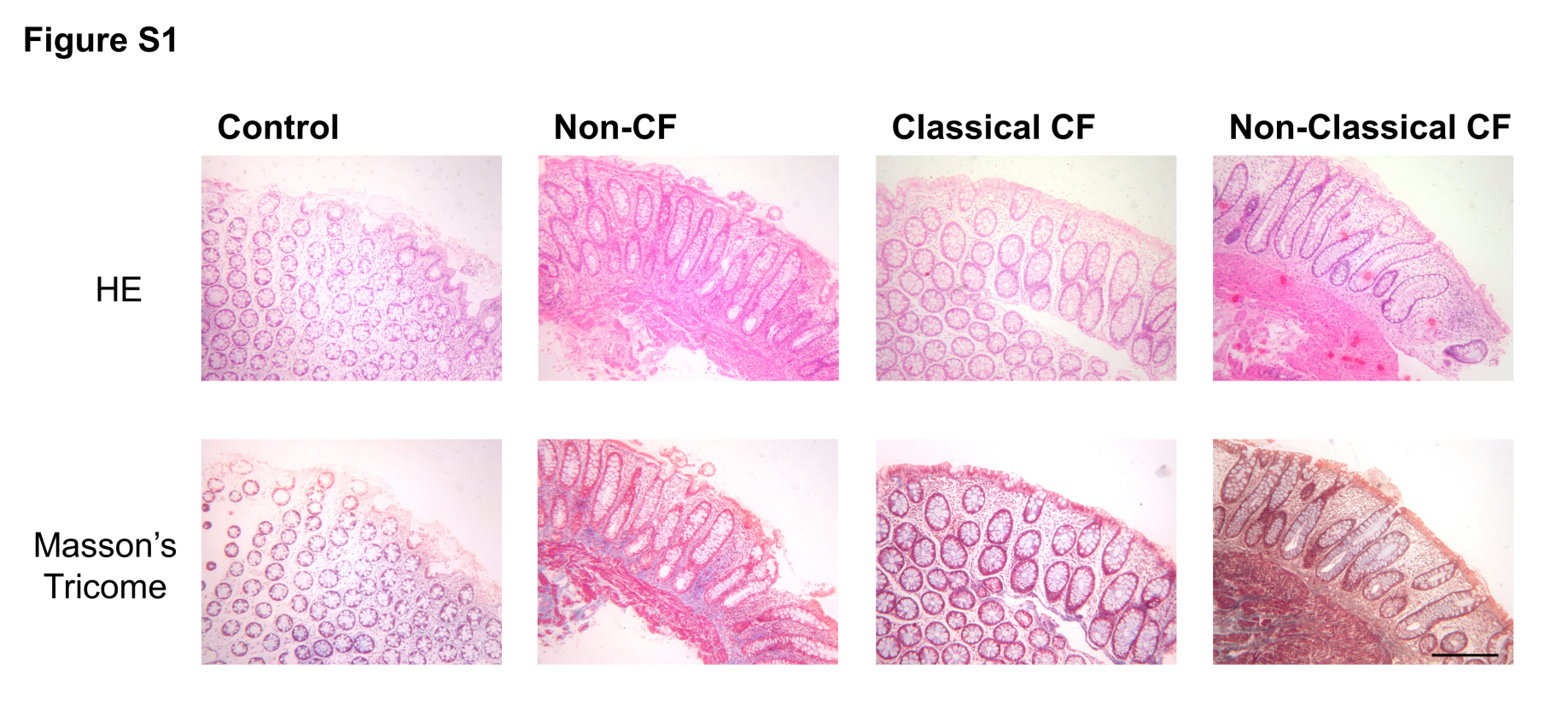
**

**Figure S1 – Histological evaluation of rectal biopsies by Hematoxilin-Eosin (HE) and Masson’s Tricome stainings** in control (transversal cut), Non-CF (longitudinal cut), Classical CF (transversal cut) and Non-Classical CF (longitudinal cut) showing a healthy epithelia, namely no fibrotic processes were observed and some biopsies presented inflammatory processes, independent of being CF or not. In HE stained sections we observe nuclei in blue and cytoplasm in pink to red. For Tricome’s Masson we observe collagen in blue, nuclei black, and muscle and cytoplasm in red. Black scale bar represents 250μM.
