# Supplementary material for: Measurements of CFTR-Mediated Cl− Secretion in Human Rectal Biopsies Constitute a Robust Biomarker for Cystic Fibrosis Diagnosis and Prognosis
Source: PLoS One. 2012 Oct 17;7(10):e47708. doi: 10.1371/journal.pone.0047708 (PMC3474728; doi:10.1371/journal.pone.0047708)
Supplement: Figure S2 — Original recordings of transepithelial voltage (Vte) measurements in Ussing chambers obtained in 3–4 rectal biopsies from the same individual evidencing the high reproducibility of the method. Rectal biopsies from (A) Non-CF individual showing large cholinergic (carbachol, CCH, 100 µM, basolateral) and cAMP-dependent (3-isobutyl-1-methylxantine, IBMX, 100 µM, and forskolin, Fsk, 2 µM, basolateral) Chloride (Cl−) secretion (lumen-negative responses); (B) CF patient homozygous for F508del-CFTR mutation with absence of Cl− secretion (only lumen-positive responses, reflecting potassium (K+) secretion, were observed); (C) CF patient (genotype: F508del/G85E-CFTR) showing very little (∼12%) cAMP-dependent Cl− secretion (biphasic responses observed upon co-cholinergic stimulation with CCH); and (D) CF patient (genotype: 3120+1G>A/L206W-CFTR) presenting larger CFTR residual function (∼57%) and milder phenotype than in (C). All the experiments were performed in the presence of Amiloride (Amil, 20 µM, luminal) and Indomethacin (Indo, 10 µM, basolateral). Transepithelial resistance (Rte) was determined from Vte deflections obtained by pulse current injection (0.5 µA). (DOCX) [file pone.0047708.s002.docx]

**
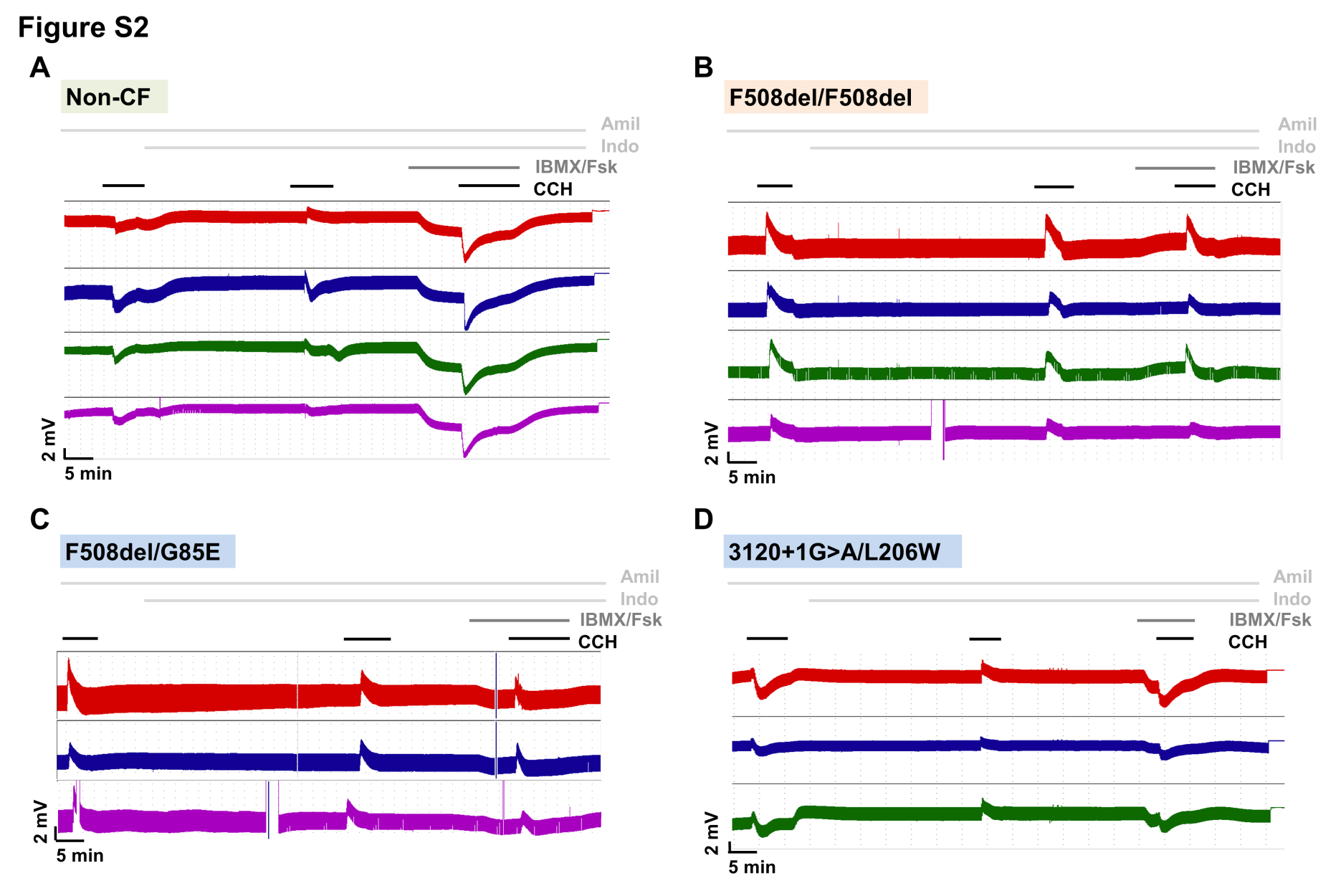
**

**Figure S2 – Original recordings of transepithelial voltage (V_te_) measurements in Ussing chambers obtained in 3-4 rectal biopsies from the same individual evidencing the high reproducibility of the method.** Rectal biopsies from **(A)** Non-CF individual showing large cholinergic (carbachol, CCH, 100 µM, basolateral) and cAMP-dependent (3-isobutyl-1-methylxantine, IBMX, 100 µM, and forskolin, Fsk, 2µM, basolateral) Chloride (Cl^-^) secretion (lumen-negative responses); **(B)** CF patient homozygous for F508del-CFTR mutation with absence of Cl^-^ secretion (only lumen-positive responses, reflecting potassium (K^+^) secretion, were observed); **(C)** CF patient (genotype: F508del/G85E-CFTR) showing very little (~12%) cAMP-dependent Cl^-^ secretion (biphasic responses observed upon co-cholinergic stimulation with CCH); and **(D)** CF patient (genotype: 3120+1G>A/L206W-CFTR) presenting larger CFTR residual function (~57%) and milder phenotype than in **(C)**. All the experiments were performed in the presence of Amiloride (Amil, 20 µM, luminal) and Indomethacin (Indo, 10 µM, basolateral). Transepithelial resistance (R_te_) was determined from V_te_ deflections obtained by pulse current injection (0.5 µA).
