# Supplementary material for: Measurements of CFTR-Mediated Cl− Secretion in Human Rectal Biopsies Constitute a Robust Biomarker for Cystic Fibrosis Diagnosis and Prognosis
Source: PLoS One. 2012 Oct 17;7(10):e47708. doi: 10.1371/journal.pone.0047708 (PMC3474728; doi:10.1371/journal.pone.0047708)
Supplement: Figure S3 — Correlations between CF clinical features and IBMX/Fsk-induced short circuit currents (Isc-IBMX/Fsk). Scatter-plot summarizing the distribution Isc-IBMX/Fsk against (A) sweat chloride concentrations (in mmol/l); (B) age at diagnosis (in years); (C) Body Mass Index distributed by groups of ages; (D) Fecal Elastase E1 concentrations (in µg/g of stools); (E) Shwachman–Kulczycki clinical scores distributed by groups of ages; and (F) FEV1 (% of predicted normal values for sex, age, and height) distributed by groups of ages. Vertical dashed black line represents subtraction of one standard deviation (STD) of the mean value calculated for Isc-IBMX/Fsk in non-CF controls (ΔIsc = −8.18 µA/cm2). Vertical dashed grey line represents addition of one STD of the mean value calculated for Isc-IBMX/Fsk in reference sub-group of Non-Classic CF patients (ΔIsc = −22.60 µA/cm2). Classic CF (filled triangles, n = 55); Non-Classic CF (filled diamonds, n = 12); CFTR-RD (star, n = 2) and Non-CF (open circles, n = 26) individuals. (DOCX) [file pone.0047708.s003.docx]

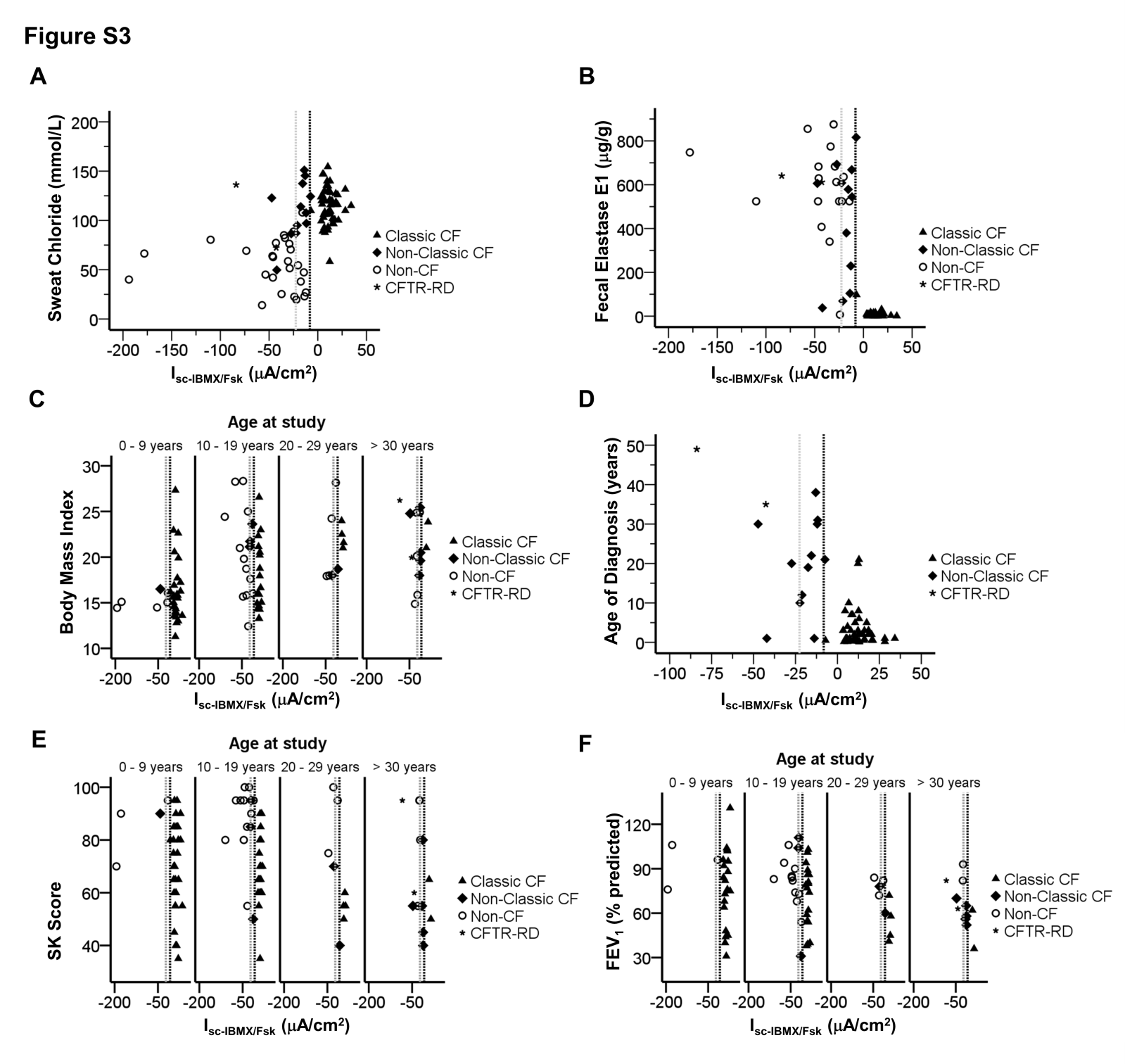


**Figure S3: Correlations between CF clinical features and IBMX/Fsk-induced short circuit currents (I_sc-IBMX/Fsk_).** Scatter-plot summarizing the distribution I_sc-IBMX/Fsk_ against **(A)** sweat chloride concentrations (in mmol/l); **(B)** age at diagnosis (in years); **(C)** Body Mass Index distributed by groups of ages; **(D)** Fecal Elastase E1 concentrations (in µg/g of stools); **(E)** Shwachman–Kulczycki clinical scores distributed by groups of ages; and **(F)** FEV1 (% of predicted normal values for sex, age, and height) distributed by groups of ages. Vertical dashed black line represents subtraction of one standard deviation (STD) of the mean value calculated for I_sc-IBMX/Fsk_ in non-CF controls (ΔI_sc_ = -8.18 µA/cm^2^). Vertical dashed grey line represents addition of one STD of the mean value calculated for I_sc-IBMX/Fsk_ in reference sub-group of Non-Classic CF patients (ΔI_sc_ = -22.60 µA/cm^2^). Classic CF (filled triangles, n = 55); Non-Classic CF (filled diamonds, n = 12); CFTR-RD (star, n= 2) and Non-CF (open circles, n = 26) individuals.
