# Supplementary material for: Measurements of CFTR-Mediated Cl− Secretion in Human Rectal Biopsies Constitute a Robust Biomarker for Cystic Fibrosis Diagnosis and Prognosis
Source: PLoS One. 2012 Oct 17;7(10):e47708. doi: 10.1371/journal.pone.0047708 (PMC3474728; doi:10.1371/journal.pone.0047708)
Supplement: Figure S4 — Correlations between CF clinical features and CCH-induced short circuit currents following IBMX/Fsk application (Isc-CCH (IBMX/Fsk)). Scatter-plot summarizing the distribution of Isc-CCH (IBMX/Fsk) against (A) Body Mass Index; (A) Body Mass Index distributed by groups of ages; (C) Shwachman–Kulczycki clinical scores; and (D) forced expiratory volume in 1 second (FEV1) expressed as a percentage of predicted normal values for sex, age, and height (% predicted).Vertical dashed black line represents subtraction of one standard deviation (STD) of the mean value calculated for CCH induced-ΔIsc following IBMX/CCH application (ΔIsc = −78.77 µA/cm2) in non-CF controls. Vertical dashed grey line represents addition of one STD of the mean value calculated for CCH stimulated-ΔIsc following IBMX/CCH application (ΔIsc = −39.55 µA/cm2) in reference sub-group of Non-Classic CF patients. Classic CF (filled triangles, n = 55); Non-Classic CF (filled diamonds, n = 12); CFTR-RD (star, n = 2) and Non-CF (open circles, n = 26) individuals. (DOCX) [file pone.0047708.s004.docx]

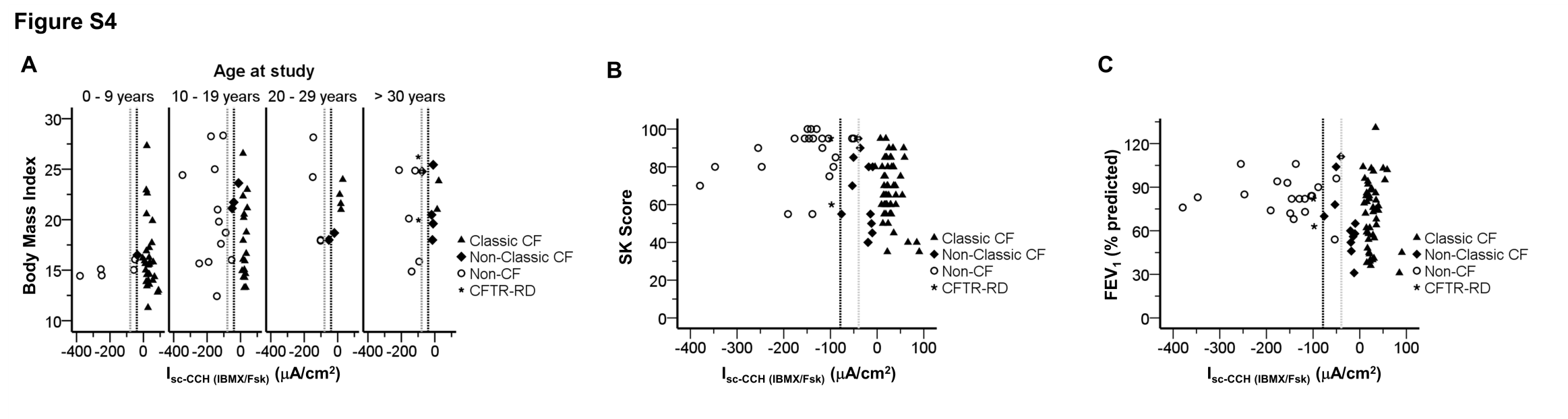
**Figure S4: Correlations between CF clinical features and CCH-induced short circuit currents following IBMX/Fsk application (I_sc-CCH (IBMX/Fsk)_).** Scatter-plot summarizing the distribution of I_sc-CCH (IBMX/Fsk)_ against **(A)** Body Mass Index; **(A)** Body Mass Index distributed by groups of ages; **(C)** Shwachman–Kulczycki clinical scores; and **(D)** forced expiratory volume in 1 second (FEV_1_) expressed as a percentage of predicted normal values for sex, age, and height (% predicted).Vertical dashed black line represents subtraction of one standard deviation (STD) of the mean value calculated for CCH induced-ΔI_sc_ following IBMX/CCH application (ΔI_sc_ = -78.77 µA/cm^2^) in non-CF controls. Vertical dashed grey line represents addition of one STD of the mean value calculated for CCH stimulated-ΔI_sc_ following IBMX/CCH application (ΔI_sc_ = -39.55 µA/cm^2^) in reference sub-group of Non-Classic CF patients. Classic CF (filled triangles, n = 55); Non-Classic CF (filled diamonds, n = 12); CFTR-RD (star, n= 2) and Non-CF (open circles, n = 26) individuals.
