# Supplementary material for: Measurements of CFTR-Mediated Cl− Secretion in Human Rectal Biopsies Constitute a Robust Biomarker for Cystic Fibrosis Diagnosis and Prognosis
Source: PLoS One. 2012 Oct 17;7(10):e47708. doi: 10.1371/journal.pone.0047708 (PMC3474728; doi:10.1371/journal.pone.0047708)
Supplement: Table S2 — CFTR mutations found in individuals under study. Gene and protein localization, mutation classification and frequency from the present study are designated. Traditional and HGVS standard nomenclature for CFTR mutations are also indicated. (DOCX) [file pone.0047708.s006.docx]

**Table S2. CFTR mutations found in individuals under study. Gene and protein localization. mutation classification and frequency from the present study are designated. Traditional and HGVS standard nomenclature^a^ for CFTR mutations are also indicated.**

| **Traditional Nomenclature** | **HGVS Nomenclature^a^** | | **Localization (CFTR gene)^d^** | **Consequence** | **Protein localization** | **Frequency (%)** | **Mutation Classification** | **Predicted Functional Class** |
| --- | --- | --- | --- | --- | --- | --- | --- | --- |
|  | **cDNA name** | **Protein name** |  |  |  |  |  |  |
| **F508del** | c.1521_1523delCTT | p.Phe508del | Exon 10 | Point deletion | NBD1 | 63.89 | A | II |
| **G542X** | c.1624G>T | p.Gly542X | Exon 11 | Nonsense | NBD1 | 7.64 | A | I |
| **R1162X** | c.3484C>T | p.Arg1162X | Exon 19 | Nonsense | ICL4 | 2.08 | A | I |
| **R334W** | c.1000C>T | p.Arg334Trp | Exon 7 | Missense | TM6 | 2.08 | A | IV |
| **3120+1G>A** | c.2988+1G>A | - | IVS16 | Splicing | - | 2.08 | A | I |
| **W1282X** | c.3846G>A | p.Trp1282X | Exon 20 | Nonsense | NBD2 | 1.39 | A | I |
| **P205S** | c.613C>T | p.Pro205Ser | Exon 6a | Missense | TM3 | 1.39 | A | IV |
| **1716+18672A>G** | c.1584+18672A>G^b^ | - | IVS 10 | Splicing^c^ | - | 1.39 | A | V |
| **1717-1G>A** | c.1585-1G>A | - | IVS11 | Splicing | - | 1.39 | A | I |
| **1812-1G>A** | c.1680-1G>A | - | IVS12 | Splicing | - | 1.39 | A | I |
| **2183AA>G** | c.2051_2052delAAinsG | p.Lys684SerfsX38 | Exon 13 | Frameshift | RD | 1.39 | A | I |
| **A561E** | c.1682C>A | p.Ala561Glu | Exon 12 | Missense | NBD1 | 1.39 | A | II |
| **3272-26A>G** | c.3140-26A>G | - | IVS17b | Splicing | - | 0.69 | A | V |
| **G85E** | c.254G>A | p.Gly85Glu | Exon 3 | Missense | TM1 | 0.69 | A | II |
| **I618T** | c.1853T>C | p.Ile618Thr | Exon 13 | Missense | NBD1/ RD | 0.69 | A | IV |
| **N1303K** | c.3909C>G | p.Asn1303Lys | Exon 21 | Missense | NBD2 | 0.69 | A | II |
| **R1066C** | c.3196C>T | p.Arg1066Cys | Exon 17b | Missense | ICL4 | 2.08 | A | II |
| **R553X** | c.1657C>T | p.Arg553X | Exon 11 | Nonsense | NBD1 | 0.69 | A | I |
| **S549R(T>G)** | c.1647T>G | p.Ser549Arg | Exon 11 | Missense | NBD1 | 0.69 | A | III |
| **V562I** | c.1684G>A | p.Val562Ile | Exon 12 | Missense | NBD1 | 0.69 | B |  |
| **L206W** | c. 617T>G | p.Leu206Trp | Exon 6a | Missense | TM3 | 1.39 | A / B | IV |
| **4428insGA** | c.4296_4297insGA | p.Ser1435GlyfsX14 | Exon 24 | Frameshift | C-terminus | 0.69 | B | VI |
| **D1152H** | c.3454G>C | p.Asp1152His | Exon 18 | Missense | TM12/NBD2 | 0.69 | B / A | IV |
| **G576A** | c.1727G>C | p.Gly576Ala | Exon 12 | Missense/Splicing | NBD1 | 0.69 | B / A | V |
| **IVS8-5T** | - | - | Exon 9 | Exon 9 skiping | - | 2.78 | B / C |  |

NOTE: A - CF-causing mutation; B - CFTR-RD mutation; C - Mutation with no clinical consequence.

^a^ Reference CFTR sequence accession number: NM_000492.3, nucleotide number 1 corresponds to the A of the ATG translation initiation codon, in the reference sequence is numbered as 133.

^b^ According to the HVGS guidelines this mutation should be named: 1585-9412bp A>G.

^c^ Inclusion of 104bp criptic exon between exon 10 and exon 11 in the CFTR transcripts.

^d^ Traditional Nomenclature.
