# Supplementary material for: Measurements of CFTR-Mediated Cl− Secretion in Human Rectal Biopsies Constitute a Robust Biomarker for Cystic Fibrosis Diagnosis and Prognosis
Source: PLoS One. 2012 Oct 17;7(10):e47708. doi: 10.1371/journal.pone.0047708 (PMC3474728; doi:10.1371/journal.pone.0047708)
Supplement: Table S3 — Mean and standard error of the mean values for clinical parameters among the 3 groups of individuals analyzed in this study: Classic CF; Non-Classic CF and Non-CF. (DOCX) [file pone.0047708.s007.docx]

**Table S3. Mean and standard error of the mean values for clinical parameters among the 3 groups of individuals analyzed in this study:**

**Classic CF; Non-Classic CF and Non-CF.**

|  |  |  |  | | **CF Clinical Diagnosis** | | | | | | | | |
| --- | --- | --- | --- | --- | --- | --- | --- | --- | --- | --- | --- | --- | --- |
|  | **Classic CF**  (I_sc-CCH(IBMX/Fsk)_ = 28.68±2.22 µA/cm^2^; n=55) | | | | | **Non-Classic CF**  (I_sc-CCH(IBMX/Fsk)_ = -30.06±6.15 µA/cm^2^; n=12) | | | | **Non-CF**  (I_sc-CCH(IBMX/Fsk)_ = -153.38±15.33 µA/cm^2^; n=28) | | | |
| **Sweat Cl^-^ (mmol/l)** | 114.23 ± 2.33 | | | | | 109.80 ± 8.34 | | | | 58.84 ± 5.38 | | | |
| **FE E1 (µg/g)** | 9.08 ± 2.04 | | | | | 444.57 ± 78.05 | | | | 585.44 ± 44.99 | | | |
| **BMI** | 17.37 ± 0.52 | | | | | 21.15 ± 0.92 | | | | 19.76 ± 0.92 | | | |
| **Age at diagnosis (yrs)** | 2.7 ± 0.6 | | | | | 19.6 ± 3.4 | | | | - | | | |
|  |  | | | | |  | | | |  | | | |
| *Aged-grouped* | *0-9 yrs* | *10-19 yrs* | | - 1. *yrs* | *≥ 30 yrs* | *0-9 yrs* | *10-19 yrs* | - 1. *yrs* | *≥ 30 yrs* | *0-9 yrs* | *10-19 yrs* | - 1. *yrs* | *≥ 30 yrs* |
| **FEV1 (% predicted)** | 77 ± 6 | 72 ± 5 | | 55 ± 5 | 49 ± 13 | - | 82 ± 26 | 61 ± 9 | 60 ± 3 | 93 ± 9 | 81 ± 4 | 79 ± 4 | 80 ± 6 |
| **FVC (% predicted)** | 85 ± 6 | 79 ± 5 | | 71 ± 8 | 69 ± 22 | - | 83 ± 17 | 69 ± 15 | 74 ± 4 | 108 ± 11 | 88 ± 4 | 83 ± 7 | 84 ± 8 |
| **SK score** | 71 ± 3 | 69 ± 3 | | 56 ± 2 | 58 ± 8 | 90 | 77 ± 14 | 63 ± 12 | 55 ± 7 | 85 ± 8 | 88 ± 4 | 90 ± 8 | 80 ± 8 |
