# Supplementary material for: Measurements of CFTR-Mediated Cl− Secretion in Human Rectal Biopsies Constitute a Robust Biomarker for Cystic Fibrosis Diagnosis and Prognosis
Source: PLoS One. 2012 Oct 17;7(10):e47708. doi: 10.1371/journal.pone.0047708 (PMC3474728; doi:10.1371/journal.pone.0047708)
Supplement: Table S4 — Discriminant Functions: Eigenvalues and Wilk’s Lambda statistics. (DOCX) [file pone.0047708.s008.docx]

**Table S4 – Discriminant Functions: Eigenvalues and Wilk’s Lambda statistics.**


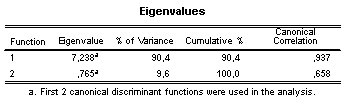


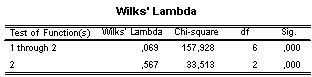


NOTE: Eigenvalues show that the biggest proportion (90.4%) of variances (in terms of differences between groups) can be explained by first discriminant function (in this case CCH-induced I_sc_ (following IBMX/Fsk application). Wilks’ Lambda (Λ) test of functions shows that both discriminant functions are significant in determining these differences between groups.
