# Supplementary material for: Measurements of CFTR-Mediated Cl− Secretion in Human Rectal Biopsies Constitute a Robust Biomarker for Cystic Fibrosis Diagnosis and Prognosis
Source: PLoS One. 2012 Oct 17;7(10):e47708. doi: 10.1371/journal.pone.0047708 (PMC3474728; doi:10.1371/journal.pone.0047708)
Supplement: Table S5 — Canonical Discriminant Functions used in the Analysis. (DOCX) [file pone.0047708.s009.docx]

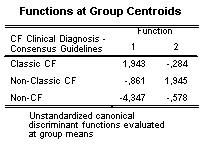

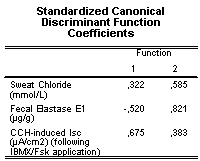

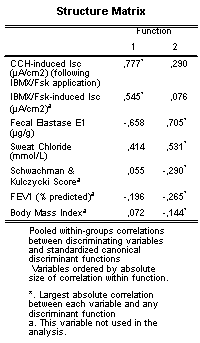
**Table S5 – Canonical Discriminant Functions used in the Analysis.**

NOTE: Structural coefficients matrix, showed on the left, explains that CCH-induced I_sc_ (following IBMX/Fsk application) is related to the first discriminant function, while Fecal Elastase E1 and Sweat Chloride values are correlated with the second discriminant function. Standardized coefficients, in the middle panel, also show the same: CCH-induced I_sc_ (following IBMX/Fsk application) is saturated for the first function, whereas both Fecal Elastase E1 and Sweat Chloride concentrations are saturated in the second one. Panel on the right shows discriminant functions at group centroids.
