# Supplementary material for: Measurements of CFTR-Mediated Cl− Secretion in Human Rectal Biopsies Constitute a Robust Biomarker for Cystic Fibrosis Diagnosis and Prognosis
Source: PLoS One. 2012 Oct 17;7(10):e47708. doi: 10.1371/journal.pone.0047708 (PMC3474728; doi:10.1371/journal.pone.0047708)
Supplement: Methods S1 — Cl- secretion in Rectal Biopsies. (DOCX) [file pone.0047708.s010.docx]

**Supplementary Methods**

*Rectal biopsies procedure*

Colon preparation (cleaning) was done by applying an enema of saline solution (0.9% NaCl) or 12% glycerine solution or by oral mannitol. Superficial 5-6 rectal mucosa specimens (3-4 mm in diameter) were obtained with or without sedation (depending on individuals’ will or collaboration) by colon forceps (Endoflex® 3.4mm, Voerde, Germany) with visual examination, avoiding the risk of bleeding or of collecting damaged tissue, and immediately stored in ice-cold RPMI1640 with 5% (v/v) Fetal Bovine Serum (FBS).

*Ussing chamber measurements*

Rectal biopsy specimens were mounted and analysed in modified micro-Ussing chambers as previously described under open-circuit conditions [1–3]. Values for the transepithelial voltage (V_te_) were referred to the serosal surface of the epithelium. Transepithelial resistance (R_te_) was determined by applying intermittent (1s) current pulses (0.5 µA). The equivalent short-circuit current (I_sc_) was calculated according to Ohm’s law (I_sc_ = V_te_ / R_te_), after appropriate correction for fluid resistance. Briefly, the luminal and basolateral surfaces of the epithelium were continuously perfused (5 ml/min) with Ringer solution of the following composition (mmol/l): NaCl 145, KH_2_PO_4_ 0.4, K_2_HPO_4_ 1.6, D-glucose 5, MgCl_2_ 1, Ca-gluconate 1.3, pH 7.40, at 37° C. HCO3^-^ free buffer solutions were used to exclude a possible contribution of CFTR-independent electrogenic HCO3^-^ secretion, which would be indistinguishable from electrogenic Cl^-^ secretion and thus may mimic residual Cl^-^ channel function in CF colonic epithelia [4]. Tissues were equilibrated in the micro-Ussing chambers for 30 min in perfused Ringer solution before the above experimental protocol [5]. Values for basal transepithelial resistance (R_te_) were similar for all groups of patients: R_te (Control)_ = 18.63 ± 0.85 Ω.cm^2^ (n = 18); R_te (non-CF)_ = 19.12 ± 0.12 Ω.cm^2^ (n = 28); R_te (Classic CF)_ = 19.60 ± 1.16 Ω.cm^2^ (n = 55); and R_te (Non-Classic CF)_ = 21.68 ± 2.80 Ω.cm^2^ (n=12). Amiloride (Amil, 20 µM, luminal) was added to block electrogenic sodium (Na^+^) absorption through the epithelial Na^+^ channel (ENaC) and Indomethacin (Indo, 10 µM, basolateral) was applied for 40-60 min to inhibit endogenous cAMP formation through prostaglandins [1–5]. As before [3,5,6], Na^+^ absorption was significantly augmented in CF rectal tissues in comparison to control (*p*=0.041) or non-CF (*p*=0.014) rectal tissues: I_sc-Amil_ _(Classic CF)_ = 86.75 ± 17.03 µA/cm^2^; I_sc-Amil_ _(Non-Classic CF)_ = 64 . 39 ± 17.79 µA/cm^2^; I_sc-Amil_ _(Control)_ = 38.56 ± 5.90 µA/cm^2^; and I_sc-Amil_ _(Non-CF)_ = 37.63 ± 6.34 µA/cm^2^. As reported [1,2] cAMP-dependent and cholinergic Cl^-^ secretion in human rectal tissues relies on functional CFTR. Thus, we used 3-isobutyl-1-methylxantine (IBMX, 100 µM, basolateral) and forskolin (2 µM, basolateral) to activate cAMP-dependent Cl^-^ secretion and carbachol (CCH, 100 µM, basolateral) for cholinergic co-activation [1–3]. Thus, percentage of CFTR function was calculated for maximal CFTR activation (ΔI_sc-IBMX/Fsk_ + ΔI_sc-CCH(IBMX/Fsk)_) and normalized to the correspondent mean value (-217.45 µA/cm^2^) for the reference non-CF control group.

*CFTR Genotyping*

To detect CFTR mutations that were not identified by screening the 6 most common CFTR mutations (F508del, G551D, G542X, R1162X, N1303K, R553X), an extended CFTR mutation search was done consisting in two-step automatic DNA sequencing of all 27 exons and the respective flanking intronic regions of the *CFTR* gene. The first step in genotyping included detection of mutations for 15 exons (exons 3, 6a, 7, 9, 10, 11, 12, 13, 16, 17b, 18, 19, 20, 21 and 24), which show a detection frequency of 95.14%. Such test method with a mutation detection rate of 95%, gives us a 90% probability of finding two abnormal alleles, 10% probability of finding one abnormal allele and 0% probability of finding no abnormal alleles [7,8]. To detect CFTR mutations that were not identified by the above described screening method, we performed DNA sequencing of the remaining 12 exons of the *CFTR* gene for CF patients. The mutations were classified according to the European Consensus [9] as: a) mutations that cause CF disease; b) mutations that result in a CFTR-related disorder; c) mutations with no known clinical consequence; and d) mutations of unproven or uncertain clinical relevance (Table S2).

*Statistics*

Pearson coefficients (*r)* were used to find correlations and partial correlations between clinical outcomes and CFTR function. As previously described [10,11], a mixed model regression analysis was chosen to determine the rate of decline in *FEV_1_ vs. Age* among the established groups; and Kruskal-Wallis test for independent samples was used to find differences between the distribution of *FEV_1_ vs. Age* across those groups. For Crosstabs, Pearson Chi-Square Tests were used to determinate independence between the variables analysed. Monte Carlo estimates of the exact p-value are provided whenever the data are too sparse or unbalanced for the asymptotic results to be reliable.

A stepwise Discriminant Analysis with Wilks’ Λ method was used to identify which variable or variables in study are able to discriminate with highest accuracy the established groups in this study. The assumptions of normality and homogeneity of variance-covariance matrices of each group were tested with Shapiro-Wilk (since one of the groups was small) and Box M tests, respectively. A Classification Analysis was also performed to obtain Fisher’s linear classification functions that could predict in which group new cases would be classified.

*Histological preparations*

Rectal biopsies collected simultaneously with the ones for bioelectrical measurements were fixed in 4% formaldehyde, embed in paraffin and cut in thin sections (2-3 µM). These sections were then deparaffinize in xylene (2 times, 10 minutes each), re-hydrate in 2 changes of absolute alcohol (5 minutes each), 95% alcohol for 2 minutes, 70% alcohol for 2 minutes and briefly in distilled water. Hematoxylin-eosin (HE) and Tricome’s Masson stainings were done as previously^6^. All slides were mounted with xylene based mounting medium. In HE stained sections we observe nuclei in blue and cytoplasm in pink to red. For Tricome’s Masson we observe collagen in blue, nuclei in black, and muscle and cytoplasm in red (blood cells in bright red).

**References**

1. Mall M, Wissner A, Seydewitz HH, Kuehr J, Brandis M, et al. (2000) Defective cholinergic Cl- secretion and detection of K+ secretion in rectal biopsies from cystic fibrosis patients. Am J Physiol Gastrointest Liver Physiol 278: G617–G624.

2. Mall M, Greger R, Seydewitz H, Al E (1998) Detection of defective cholinergic Cl- secretion in human rectal biopsies for the diagnosis of Cystic Fibrosis. J Clin Invest 102: 15–21.

3. Hirtz S, Gonska T, Seydewitz HH, Thomas J, Greiner P, et al. (2004) CFTR Cl- channel function in native human colon correlates with the genotype and phenotype in cystic fibrosis. Gastroenterology 127: 1085–1095.

4. Mall M, Kreda SM, Mengos A, Jensen TJ, Hirtz S, et al. (2004) The DF508 Mutation Results in Loss of CFTR Function and Mature Protein in Native Human Colon. Gastroenterology 126: 32–41.

5. Mall M, Hirtz S, Gonska T, Kunzelmann K (2004) Assessment of CFTR function in rectal biopsies for the diagnosis of cystic fibrosis. J Cyst Fibros 3 (S2): 165–169.

6. Mall M, Bleich M, Kuehr J, Brandis M, Greger R, et al. (1999) CFTR-mediated inhibition of epithelial Na+ conductance in human colon is defective in cystic fibrosis. Am J Physiol 277: G709–G716.

7. Moskowitz SM, Chmiel JF, Sternen DL, Cheng E, Cutting GR (2001) CFTR-related disorders. In: Pagon R, Bird T, Dolan C, Stephens K, editors. University of Washington, Seattle, WA, USA.

8. Strom CM, Huang D, Chen C, Buller A, Peng M, et al. (2003) Extensive sequencing of the cystic fibrosis transmembrane regulator gene: assay validation and unexpected benefits of developing a comprehensive test. Genet Med 5: 9–14.

9. Castellani C, Cuppens H, Macek Jr. M, Cassiman JJ, Kerem E, et al. (2008) Consensus on the use and interpretation of cystic fibrosis mutation analysis in clinical practice. J Cyst Fibros 7: 179–196.

10. Schaedel C, de MI, Hjelte L, Johannesson M, Kornfalt R, et al. (2002) Predictors of deterioration of lung function in cystic fibrosis. Pediatr Pulmonol 33: 483–491.

11. Cleveland RH, Zurakowski D, Slattery D, Colin AA (2009) Cystic fibrosis genotype and assessing rates of decline in pulmonary status. Radiology 253: 813–821.
